# Case Report: The Imperfect Association Between Craniofacial Lesion Burden and Pain in Fibrous Dysplasia

**DOI:** 10.3389/fneur.2022.855157

**Published:** 2022-03-16

**Authors:** Emma Golden, Fan Zhang, Daryl J. Selen, David Ebb, Laura Romo, Laura A. Drubach, Nehal Shah, Lauren J. O'Donnell, Jordan D. Lemme, Rachel Myers, Mariesa Cay, Henry M. Kronenberg, Carl-Fredrik Westin, Alison M. Boyce, Leonard B. Kaban, Jaymin Upadhyay

**Affiliations:** ^1^Department of Anesthesiology, Critical Care and Pain Medicine, Boston Children's Hospital and Harvard Medical School, Boston, MA, United States; ^2^Department of Radiology, Brigham and Women's Hospital and Harvard Medical School, Boston, MA, United States; ^3^Endocrine Unit, Department of Medicine, Massachusetts General Hospital, Boston, MA, United States; ^4^Harvard Medical School, Boston, MA, United States; ^5^Department of Pediatric Hematology Oncology, Massachusetts General Hospital and Harvard Medical School, Boston, MA, United States; ^6^Head and Neck Imaging, Department of Radiology, Massachusetts Eye and Ear, Harvard Medical School, Boston, MA, United States; ^7^Department of Radiology, Boston Children's Hospital and Harvard Medical School, Boston, MA, United States; ^8^Metabolic Bone Disorders Unit, National Institute of Dental and Craniofacial Research, National Institutes of Health, Bethesda, MD, United States; ^9^Department of Oral and Maxillofacial Surgery, Massachusetts General Hospital and Harvard School of Dental Medicine, Boston, MA, United States; ^10^Department of Psychiatry, McLean Hospital and Harvard Medical School, Belmont, MA, United States

**Keywords:** fibrous dysplasia, craniofacial lesions, trigeminal nerve system, pain, headache, migraine

## Abstract

Patients with fibrous dysplasia (FD) often present with craniofacial lesions that affect the trigeminal nerve system. Debilitating pain, headache, and migraine are frequently experienced by FD patients with poor prognosis, while some individuals with similar bone lesions are asymptomatic. The clinical and biological factors that contribute to the etiopathogenesis of pain in craniofacial FD are largely unknown. We present two adult females with comparable craniofacial FD lesion size and location, as measured by ^18^F-sodium fluoride positron emission tomography/computed tomography (PET/CT), yet their respective pain phenotypes differed significantly. Over 4 weeks, the average pain reported by Patient A was 0.4/0–10 scale. Patient B reported average pain of 7.8/0–10 scale distributed across the entire skull and left facial region. Patient B did not experience pain relief from analgesics or more aggressive treatments (denosumab). In both patients, evaluation of trigeminal nerve divisions (V1, V2, and V3) with CT and magnetic resonance imaging (MRI) revealed nerve compression and displacement with more involvement of the left trigeminal branches relative to the right. First-time employment of diffusion MRI and tractography suggested reduced apparent fiber density within the cisternal segment of the trigeminal nerve, particularly for Patient B and in the left hemisphere. These cases highlight heterogeneous clinical presentation and neurobiological properties in craniofacial FD and also, the disconnect between peripheral pathology and pain severity. We hypothesize that a detailed phenotypic characterization of patients that incorporates an advanced imaging approach probing the trigeminal system may provide enhanced insights into the variable experiences with pain in craniofacial FD.

## Introduction

Fibrous dysplasia (FD, OMIM 174800) is a rare bone disease arising from an R201 missense mutation of the *GNAS* gene (1–3). FD may be complicated by co-existing pigmented skin lesions, precocious puberty, and other endocrinopathies resulting in the diagnosis of McCune Albright Syndrome (MAS) (4). Pain remains a complex, inadequately understood and poorly managed feature of FD (5–7). Craniofacial skeletal lesions in FD are the probable and inciting cause of atypical facial pain, headaches, or migraines (8–10). However, the severity or type of pain reported by patients with FD is highly variable. Further complicating the understanding of pain in FD or its treatment is the weak correlation between patient-reported pain intensity and skeletal disease burden (11). This dissociation points to the need to uncover both clinical and active biological mechanisms that cause pain in individual patients with FD.

In the current comparative case report, two adult (22 years of age) female patients with similar craniofacial FD lesion burden, but who presented with contrasting pain experiences are described. However, the patients contrasted tremendously in terms of their respective experiences with pain. Over a period of many years, Patient A reported little to no pain, while Patient B has had an unfortunately long history of suffering from craniofacial pain in the trigeminal distribution, headaches, and migraines. A multidisciplinary approach was taken to investigate potential factors associated with the divergent pain profiles specific to Patients A and B. Our strategy involved phenotyping pain and related symptoms (i.e., altered mood) using several clinical instruments. In parallel, molecular imaging techniques were employed to define craniofacial lesion burden and FD's impact on trigeminal nerves and branches. Examination and comparison between the two FD patient datasets indicate the importance of the need for a closer psychological and neurological assessment of pain in FD.

## Clinical Overview

This study was approved by the Boston Children's Hospital (BCH) and the Massachusetts General Brigham, Institutional Review Boards. Patient A and Patient B provided informed consent and underwent study evaluation in January 2021 and February 2021, respectively.

### Patient A, Craniofacial FD Without Pain

Patient A is a 22-year-old woman with polyostotic FD of the left zygomaticomaxillary complex (maxilla, zygoma and sphenoid bones) and parasymphyseal regions of the mandible. Her disease was first identified at age 11 when she presented with a painless swelling of the left maxilla during a routine dental examination. Panoramic radiograph at that time demonstrated a radio-opaque lesion, particularly of the left zygomaticomaxillary complex and filling the left maxillary sinus. She was subsequently referred to oral and maxillofacial surgery (OMS) at Massachusetts General Hospital (MGH) where a computed tomographic (CT) scan of the facial bones revealed marked expansion of the left maxilla, zygoma, and sphenoid bones with a ground glass appearance, consistent with a diagnosis of FD. This was confirmed by histologic evaluation of a biopsy specimen in August 2010.

Following this initial consultation in OMS, further evaluations were undertaken by Ophthalmology, Endocrinology, Pediatric Orthopedic Surgery, and Neuro-ophthalmology to document the presence of additional osseous pathology and/or functional deficits. A Tc-99m-methylene diphosphonate technetium single-photon emission computerized tomography (SPECT) scan in August 2010 revealed no other foci of FD. Skin examination demonstrated a small hyperpigmented lesion over the lower back measuring 1 × 2 cm. Serial endocrine evaluations have demonstrated no evidence of precocious puberty or other endocrinopathies that might be present in MAS. Despite narrowing of the optic canal and displacement of her left globe by growth of the bone lesions in the left sphenoid wing, zygoma, and maxilla, her visual acuity has remained normal during serial examinations, with no evidence of visual field defects or optic nerve pathology.

During 11 years of follow-up, the patient has undergone multiple contour resections of the left facial skeleton to manage progressive expansile changes in the left maxilla, zygoma, and sphenoid bones with associated exophthalmos. The first procedure was performed in August 2012, at 13.5 years of age, when she presented with expansile changes in the left zygomaticomaxillary complex, proptosis and intermittent mild tenderness over the corresponding left side of the face. Since the initial operation, she has not had pain or tenderness despite intermittent growth of the FD warranting 3 additional contour resections: June 2012, December 2015, and May 2018. During the 3-year period since 2018, the disease has been quiescent with only slow, minor growth. Based on this history, finishing college and entering the workforce, she had, what is hoped will be, a final contour resection of the zygomaticomaxillary lesion in June 2021.

### Patient B, Craniofacial FD With Pain

Patient B was first diagnosed with polyostotic craniofacial FD in November 2019 at age 21. She initially presented at that time with worsening of her chronic headaches, vision changes, amnesia, and gait imbalance. She reported fatigue for the prior month, worsening headaches for 2–3 weeks, blurry vision for 2–3 days, and a week of forgetfulness that culminated in her getting lost while driving, prompting presentation to an emergency department (ED). In retrospect, she had noticed severe headaches for the past 5 years and had a history of four concussions from 2014 to 2017 from motor vehicle accidents and playing soccer. At the outside hospital, she had a cranial CT that revealed a sclerotic left skull lesion involving the left sphenoid wing, anterior clivus, left pterygoid plate, and left temporal bone, so she was transferred to the MGH ED for further evaluation. Imaging was repeated at MGH and CT revealed ground glass marrow expansion of the sphenoid bone and left temporal bone consistent with FD with narrowing of the skull base foramina, fissures, and middle cranial fossa. Endocrinology was consulted while the patient was in the ED due to new diagnosis of FD, as well as concern for pituitary compression and need for a hormonal evaluation.

During her initial evaluation in the ED, her calcium, phosphate, magnesium, parathyroid hormone, albumin, and 25-OH vitamin D labs were normal. On exam, she had no café-au-lait skin lesions or history of precocious puberty or hyperfunctioning endocrinopathies, so the clinical suspicion for MAS was low. Additional endocrine evaluation revealed normal thyroid function, no evidence of adrenal insufficiency, and otherwise normal gonadal and insulin-like growth factor-I hormonal axes. OMS and Neuro-ophthalmology recommended no surgical treatment due to normal visual exam and auditory testing. For her daily severe, debilitating headaches, the patient was further evaluated by Neurology, and her headaches were attributed to the FD of the skull, with an additional component of intractable migraine without aura. Patient B was also diagnosed with trigeminal neuralgia. For headaches, she achieved no symptomatic relief with magnesium, riboflavin, sumatriptan, topiramate, or ibuprofen. She was treated with On abotulinumtoxinA injections for migraines in August 2020, July 2021, and November 2021, and she did experience temporary symptomatic relief for a few weeks after each treatment.

Due to her desire for pregnancy within a few years after diagnosis, treatment with denosumab was recommended instead of bisphosphonates due to concerns of long-lasting retention of bisphosphonates in the bones and possible future fetal exposure (12). Denosumab, a monoclonal antibody to RANK ligand that directly inhibits osteoclastogenesis, was used off-label to attempt to reduce pain associated with FD as well as lesion expansion (13–18). She received two 120 mg doses of denosumab in total spaced by 6 months in 2020 but did not notice any improvement in her pain. This was in contrast to prior reports noting fast analgesic action following treatment induction with denosumab. Notably, there has not been expansion of her FD lesions in the year since her last dose of denosumab. The patient was further discussed with Neurosurgery and Radiation Oncology who thought that neither debulking nor radiation to the areas of FD were easy or safe options due to the risk of significant complications. She continues to receive Botox injections for headache and migraine relief, but otherwise has not achieved symptomatic control of her headaches or migraines.

## Clinical Pain

**Figure 1:**
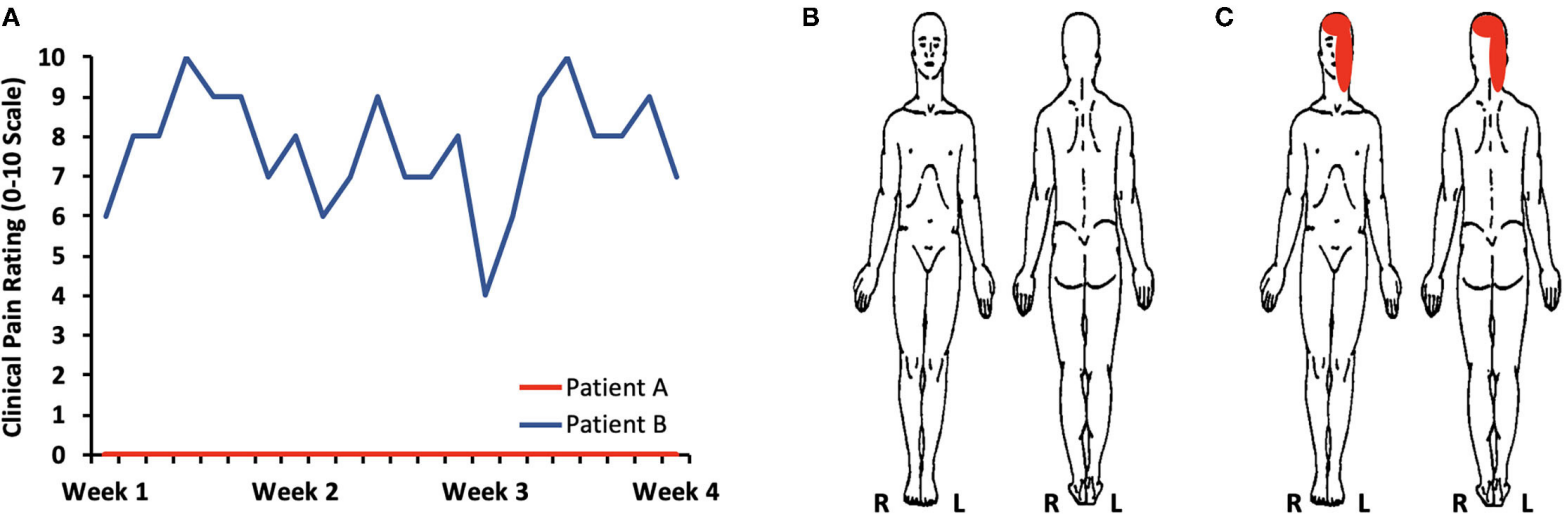
Clinical pain levels. **(A)** Self-reported daily pain levels over 4 weeks confirms the presence of little to no pain for Patients A (average pain: 0.4/0–10 scale) and moderate to severe pain for Patient B (average pain: 7.8/0–10 scale). **(B)** A body map further defined an absence of pain in Patient A. **(C)** Patient B described pain primarily on the left side of her face and over the majority of the skull.

Patient A not only reported a minimal amount of clinical pain, but also showed low levels of psychological symptoms or distress (Supplementary Table 1). Self-reported daily pain levels monitored over 4 weeks confirmed the absence or minimal amount of pain for Patient A (Figure 1). In contrast, Patient B harbored severe pain, consistent with her experience of daily headaches with superimposed migraines in addition to intermittent trigeminal neuralgia. High levels of craniofacial and headache-type pain were present at the time of imaging evaluation (Supplementary Table 1) as well as over the course of 4 weeks (Figure 1). In conjunction with experiencing severe pain, Patient B presented with a clinically relevant level of pain catastrophizing–a maladaptive cognitive and emotional response to pain (19, 20). The presence of pain catastrophizing was in line with the occurrence psychological stress with mild levels of depressive symptoms. Patients A and B reported similar levels of sleep quality.

## Craniofacial Lesion Burden and Severity

**Figure 2:**
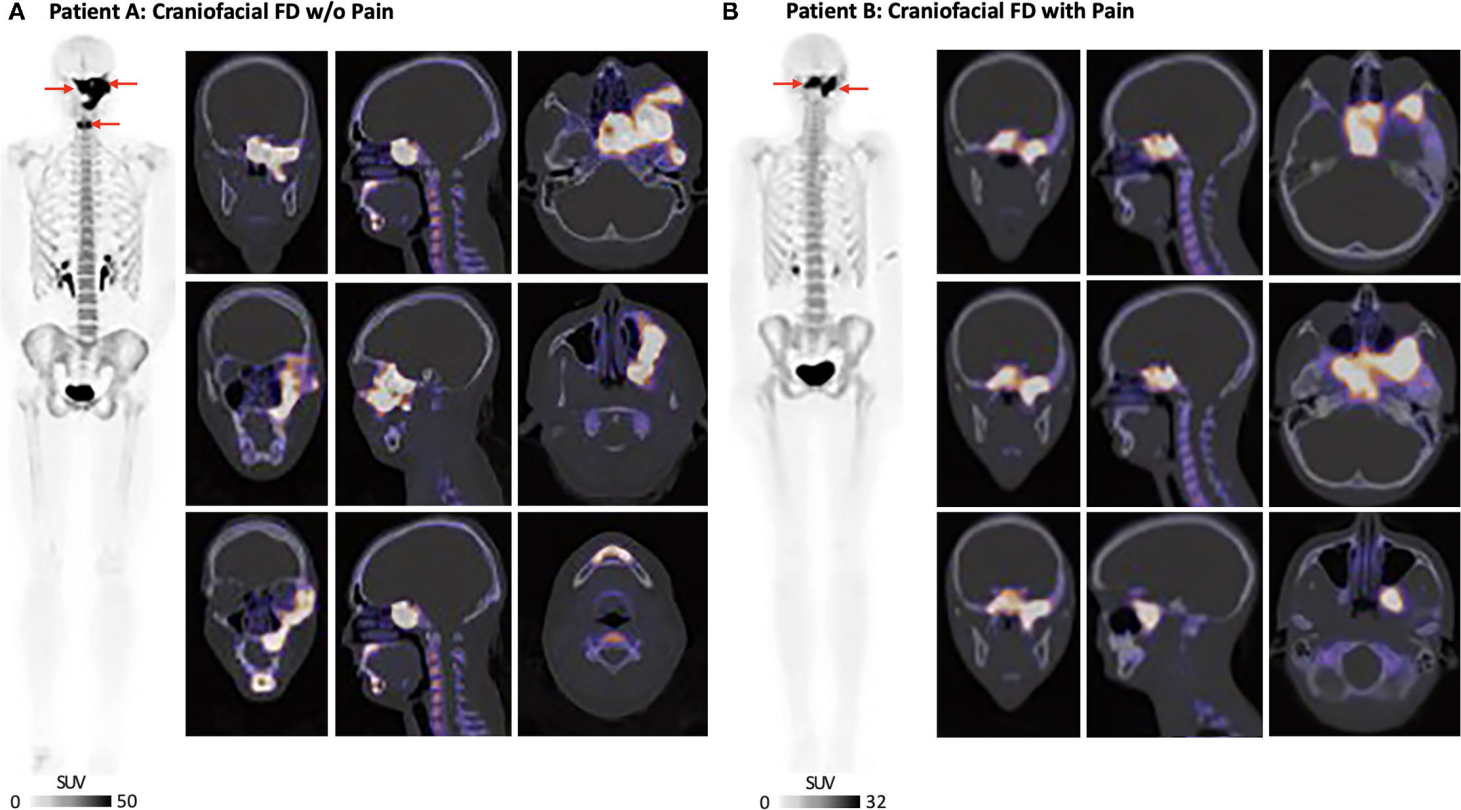
Craniofacial FD burden and severity. **(A)** Patient A: ^18^F-NaF PET/CT showed intense radiotracer uptake in the left frontal bone and zygomatic arch, left maxilla, left sphenoid bone, and livus. There was also involvement of the anterior mandible. **(B)** Patient B: ^18^F-NaF PET/CT showed intense uptake involving the base of the skull, clivus, sella and left sphenoid bone. The patient also had scattered areas of increased uptake in the left temporal bone with SUV_max_ = 13. All ^18^F-NaF PET/CT data were read by a board-certified nuclear medicine physician (Dr. Drubach).

Whole-body ^18^F-sodium fluoride positron emission tomography/computed tomography [^18^F-NaF PET/CT; Injected dose = 4.0 millicurie (mCi) of ^18^F-NaF] was performed at BCH and on a Siemens Biograph Vision system (Siemens, Erlangen, Germany). In Patient A, whole-body ^18^F-NaF PET/CT showed intense radiotracer uptake corresponding to ground glass and expansile appearance of the left inferolateral frontal bone involving the ipsilateral zygoma and zygomatic arch (Figure 2). Uptake of ^18^F-NaF was evident in the sphenoid bone (involving both clinoid processes) and clivus extending across midline, as well as the left pterygoid plates and the left maxilla extending to the midline alveolar ridge and surrounding the left-sided maxillary incisors, bicuspids, and molars. There was a separate but discrete area of intense radiotracer uptake along the parasymphyseal mandible along the anterior aspect. Scattered areas of lytic-appearing bone were noted in the maxilla and left sphenoid wing.

Whole-body ^18^F-NaF PET/CT (Injected dose = 4.0 mCi of ^18^F-NaF) performed in Patient B showed overlapping craniofacial FD burden relative to Patient A. Several regions of increased ^18^F-NaF uptake in the skull were consistent with FD involvement in Patient B (Figure 2). There was intense uptake in the midline of the base of the skull involving the clivus and sella, left sphenoid bone, and most medial regions of the right sphenoid bone. There were a few scattered areas of increased uptake in the left temporal bone. The areas of increased uptake were associated with expanded ground glass appearance of the bone on CT. ^18^F-NaF uptake outside of the craniofacial regions was not observed for either Patients A or B.

## The Impact of Craniofacial FD on Trigeminal Nerves

**Figure 3:**
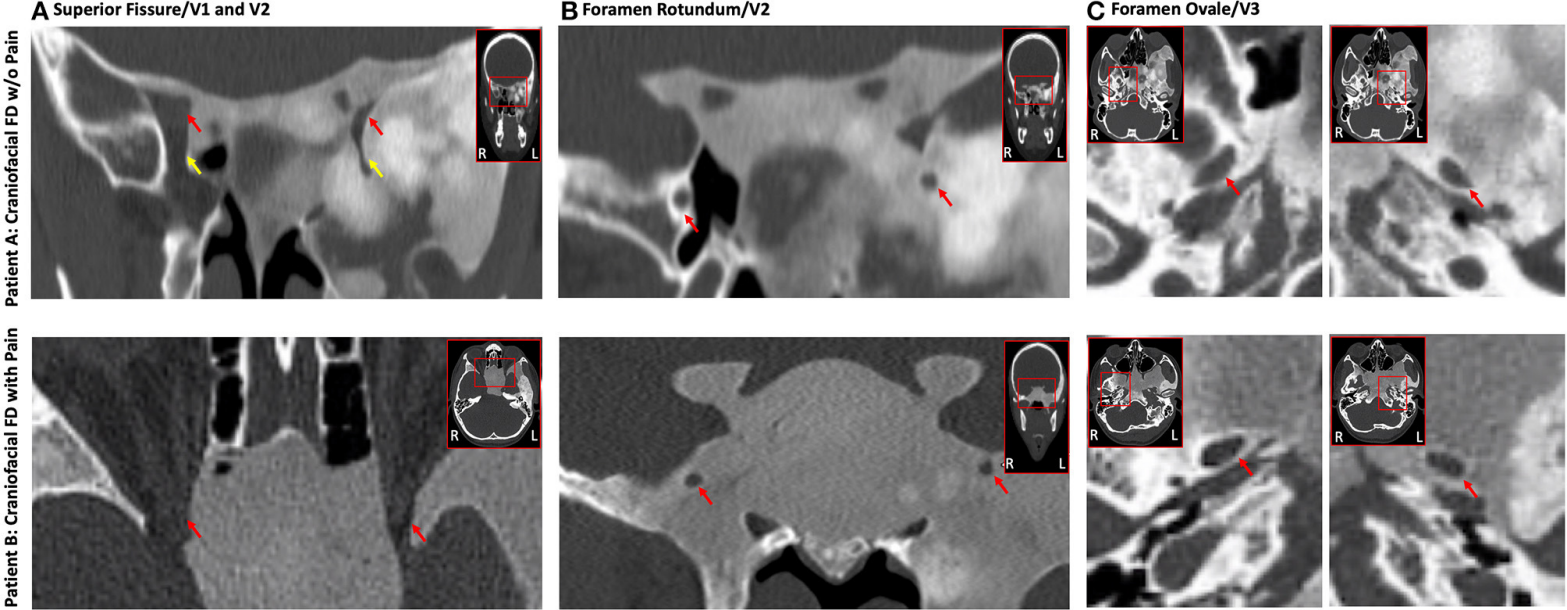
Impact of FD on the trigeminal nerve system. Craniofacial FD lesions caused a highly similar impact on conduits carrying trigeminal nerves in both Patients A and B. Displacement and narrowing of the V1 **(A)**, V2 **(B)**, and V3 **(C)** divisions of the trigeminal nerves was evident on CT for both FD patients with generally a greater impact on left hemisphere structures relative to the right. The top (Patient A) and bottom (Patient B) rows show coronal or axial cross-sections from CT. **(A)** Constriction of the superior orbital fissure (red arrow) and inferior orbital fissure (yellow arrow; Patient A only). **(B)** Narrowing and displacement of the left foramen rotundum (red arrows). **(C)** Narrowing and displacement of the left foramen ovale (red arrows). **(C)** All data were read by a board-certified neuroradiologist (Dr. Romo).

Non-contrast magnetic resonance imaging (MRI) was performed on a 3T Siemens Prisma with a 64-channel head coil (Siemens, Erlangen) at McLean Hospital. For both Patients A and B, involvement of the sphenoid bone by FD resulted in a mass effect as well as narrowing and displacement of the left superior fissure, which transmits the first (V1) division of the left trigeminal nerve (CNV), the left foramen rotundum (FR) transmitting the second (V2) division of CNV, and the left foramen ovale (FO) transmitting the third (V3) division of CNV (Figure 3). In addition, there was narrowing of the left superior orbital fissure (SOF). Overall and for both patients, the narrowing and displacement was more prominent for left hemisphere cranial structures relative to the right. Patient A additionally showed structural alterations of the left inferior orbital fissure and the left infraorbital canal, both of which transmit the infra-orbital nerve, a branch of V2. More proximally, FR and FO were narrowed and superolaterally displaced on the left compared to the right.

**Figure 4:**
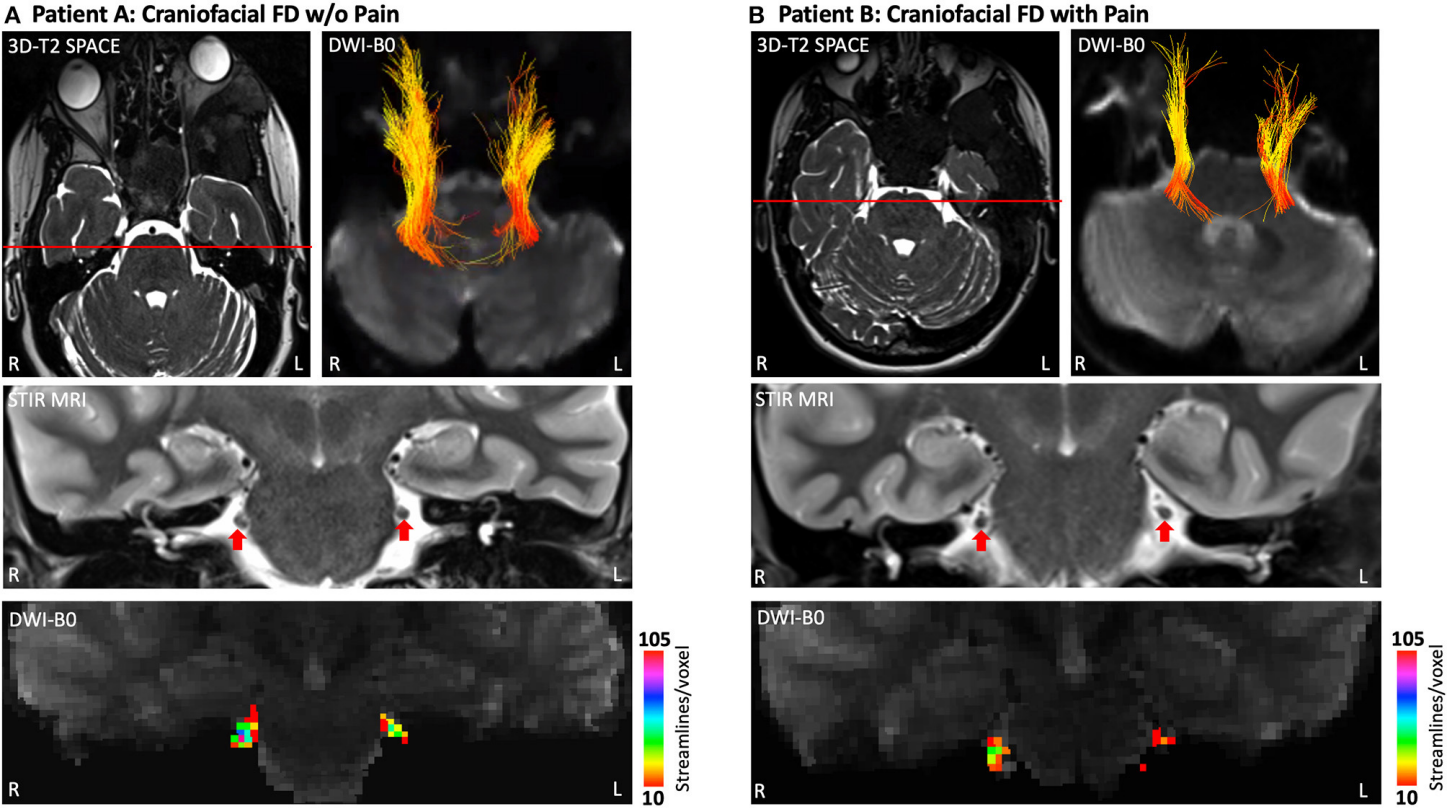
Apparent fiber loss in the CNV (cisternal segment). Patient A **(A)** and Patient B **(B)** each demonstrated a lower level of apparent fiber density in left hemisphere CNV at the cisternal level as determined by diffusion MRI and tractography. Fiber streamlines projecting between the brainstem and trigeminal ganglion are shown colored by fractional anisotropy (FA). Coronal cross-sections of T2-SPACE MRI show normal caliber and symmetry of CNV for both patients. However, heat maps derived from diffusion tractography indicate a lower magnitude of streamlines per voxel (apparent fiber density) for the left hemisphere CNV, particularly for Patient B. Heat maps for the right hemisphere CNV indicate a lower level of streamlines per voxel in Patient B compared to Patient A. DTI data were collected using Human Connectome Project protocols (29). 3D-T2-SPACE, 3D-T2 Sampling Perfection with Application-Optimized Contrasts by Using Flip Angle Evolution; FA, Fractional Anisotropy; STIR MRI, Short Tau Inversion Recovery Magnetic Resonance Imaging; DWI, Diffusion Weighted Imaging.

Using a combination of diffusion MRI and tractography, the apparent axonal fiber density of the cisternal segment of CNV (brainstem to trigeminal ganglion) was investigated with previously described procedures (21, 22) (Figure 4). Although the caliber and symmetry of CNV on T2-SPACE MRI and STIR MRI appeared normal for Patients A and B, both individuals appeared to have a of loss apparent fiber density in the left CNV relative to the right. However, Patient B appeared to differentiate from Patient A based on a comparatively lower level of bilateral fiber density (defined as the number of streamlines/voxel). The latter novel finding suggests modulation of trigeminal system function and structure, and possibly involvement of central pain processing pathways in FD. Further investigation may demonstrate the utility of diffusion MRI to more directly document nerve pathology in patients with craniofacial FD.

## Discussion

The two cases described in the current report point to the remarkable spectrum of pain phenotypes present in FD. On one hand, the heterogeneous nature of FD likely contributes to the variability of both the pain severity reported across patients, as well as the responses to palliative treatments that aim to manage FD lesions or mitigate pain (e.g., reconstructive surgery, On abotulinumtoxinA injections, bisphosphonate, denosumab, and analgesics) (13–17, 23–26). Yet Patients A and B possessed a number of similarities from demographic (i.e., age and gender) and biological (i.e., anatomical distribution plus severity of craniofacial lesions and non-MAS status) perspectives. Considering the many parallels between the two FD patients, the extreme differences regarding pain is perplexing, but also highlights the challenge in identifying how best clinically to approach pain treatment in FD. Moreover, adding to the complexity in providing effective pain treatment to patients with FD is the high probability of many unknown clinical and biological drivers of pain active in individual patients or possibly, FD subtypes.

Patients A and B did in fact differentiate in multiple domains, which may suggest why Patient B currently experiences craniofacial pain and migraines headaches. Diagnosis of FD for Patients A and B occurred at ages 11 and 21, respectively. Thus, the implementation of surgical care and other treatments in Patient A much earlier in the course of disease as well as (neuro-) development may have provided the foundation necessary to limit, for example, maladaptive neurological process associated with peripheral or central pain sensitization. It is noted, however, that many patients with FD/MAS have an even earlier diagnosis and receive treatment shortly after yet still suffer from pain, headaches or migraine. Patient B not only had a much later FD diagnosis, but also experienced multiple concussions, which collectively may have contributed to or worsened her chronic craniofacial pain and migraine phenotype (27, 28). Further insights into why Patient B has not experienced pain relief despite the use of multiple modes of therapy may be garnered by considering her feelings of helplessness or constant rumination of pain as defined by the pain catastrophizing scale. An additional possibility is that long-term suffering from almost constant headaches and migraines may have caused her to catastrophize about pain and become psychologically distressed. Importantly, such a robust emotional state may solidify the presence of pain and drive some patients with FD to become resistant to various analgesic treatment strategies. Finally, Patients A and B also differed according to how their respective craniofacial lesions structurally impacted trigeminal nerves and branches. In both patients, CT and MRI demonstrated narrowing and displacement of V1, V2, and V3; an effect that appeared more notable on left hemisphere craniofacial structures compared to the right. Diffusion tractography revealed abnormalities in the cisternal segment of the trigeminal nerve, where the loss of apparent fiber density was more notable for Patient B.

Based on the current comparative case study, we project that in a sub-population of patients with craniofacial FD, a combination of neurological abnormalities, particularly those more proximal to the central nervous system, together with the psychological aspects of pain facilitate a complex and difficult to treat pain state. Whether and how peripheral and central features drive pain in FD should be the focus of future prospective studies, which may result in identification of biomarkers in FD/MAS that can predict pain trajectories, prognosis, or treatment response.

## Data Availability Statement

The data that support the findings of this study are available upon reasonable request.

## Ethics Statement

The studies involving human participants were reviewed and approved by Boston Children's Hospital IRB. The patients/participants provided their written informed consent to participate in this study. Written informed consent was obtained from the individual(s) for the publication of any potentially identifiable images or data included in this article.

## Author Contributions

EG, FZ, LR, LD, LO'D, and C-FW analyzed data, drafted the initial manuscript, and reviewed and revised the manuscript. DS, DE, HK, and LK provided patient care, acquired and analyzed data, drafted the initial manuscript, and reviewed and revised the manuscript. NS, JL, RM, and MC designed the study and acquired and analyzed data. AB designed the study and drafted the initial manuscript. AB and JU acquired funding in support of this case report. JU designed the study, acquired and analyzed data, drafted the initial manuscript, and reviewed and revised the manuscript. All authors approved the final manuscript as submitted and agree to be accountable for all aspects of the work.

## Funding

This research was funded by the MAYDAY Fund (PI: JU). DS was supported by NIH T32 grant (T32DK007028).

## Conflict of Interest

AB through the National Institute of Dental and Craniofacial Research, receives support from Amgen, Inc., for an investigator sponsored study of denosumab treatment for fibrous dysplasia. The remaining authors declare that the research was conducted in the absence of any commercial or financial relationships that could be construed as a potential conflict of interest. The reviewer A-MH declared a past co-authorship with one of the authors AB to the handling Editor.

## Publisher's Note

All claims expressed in this article are solely those of the authors and do not necessarily represent those of their affiliated organizations, or those of the publisher, the editors and the reviewers. Any product that may be evaluated in this article, or claim that may be made by its manufacturer, is not guaranteed or endorsed by the publisher.
