# Supplementary material for: Case Report: The Imperfect Association Between Craniofacial Lesion Burden and Pain in Fibrous Dysplasia
Source: Front Neurol. 2022 Mar 16;13:855157. doi: 10.3389/fneur.2022.855157 (PMC8966612; doi:10.3389/fneur.2022.855157)
Supplement: Supplementary file 1 [file Table_1.DOCX]

| Patient | Age (y) | Gender | BPI  Worst Pain  Least Pain  Average Pain  Pain Right Now | BPI: Pain Descriptors | Pain Catastrophizing Scale;  Total  Rumination  Magnification  Helplessness | Allodynia | Photophobia (y/n) | Hospital Anxiety & Depression Scale  Depression  Anxiety | Perceived Stress  Scale | Pittsburgh Sleep Quality Index |
| --- | --- | --- | --- | --- | --- | --- | --- | --- | --- | --- |
| Patient A | 22 | F | 1  1  1  0 | Aching, Nagging | 0  0  0  0 | 0 | n | 1  0  1 | 6 | 11 |
| Patient B | 22 | F | 10  6  8  8 | Aching, Shooting, Stabbing, Sharp,  Exhausting, Tiring, Penetrating, Nagging, Miserable, Unbearable | 38  15  6  17 | 1 | Y  (baseline &  during headaches) | 11  4  7 | 22 | 14 |

**Brief Pain Inventory (BPI)**

0-10 Scale

Worst Pain 🡪 Over the last 7 days

Least Pain 🡪 Over the last 7 days

Average Pain 🡪 No time scale

**Pain Catastrophizing Scale (PCS)**

Total score ranging from 0-52

Total score of 30+ indicates clinically relevant level of catastrophizing

Subscale score range

PCS Rumination: 0-16

PCS Magnification: 0-12

PCS Helplessness: 0-24

**Allodynia Symptom Checklist**

None: 0‐2

Mild: 3‐5

Moderate: 6‐8

Severe: 9 or more

**Hospital Anxiety and Depression Scale (HADS)**

Total score ranging from 0-21 for each HADS subscale

0-7: Normal

8-10: Borderline abnormal

11-21: Abnormal

**Perceived Stress Scale**

Total score ranging from 0-40

0-13: Low stress

14-26: Moderate stress

27-40: High perceived stress

**PSQI: Pittsburgh Sleep Quality Index**

Total score ranging from 0-21

Higher scores correspond to poorer sleep quality
